# Airway Tube Exchanger Techniques in Morbidly Obese Patients

**DOI:** 10.1155/2012/968642

**Published:** 2012-02-09

**Authors:** Danai Udomtecha

**Affiliations:** Department of Anesthesia, University of Iowa Hospitals and Clinics, Iowa City, IA 52242, USA

## Abstract

Morbidly obese patients may present a challenge during airway management. When airway tube exchange is required, it can even be more challenging than the primary intubation. With the increasing prevalence of morbid obesity over the years, there will be increasing numbers of these patients presenting for surgical procedures, including ones that require endotracheal tube exchanges. It is therefore important for anesthesiologists to be familiar with options and limitations of the airway tube exchanger techniques.

## 1. Introduction

Obesity has become a public health crisis in the United States. The prevalence of obesity doubled between 1976–1980 and 1999-2000, increasing from 15.1 percent to 30.9 percent [1]. Results from the 2007-2008 National Health and Nutrition Examination Survey (NHANES) indicate that an estimated 34.2% of US adults aged 20 years and over are overweight, 33.8% are obese, and 5.7% are extremely obese [2]. With the prevalence of obesity and morbid obesity on the rise, all healthcare specialties will see more and more of these patients. There will be increasing numbers of obese patients presenting for surgical procedures, including ones that require endotracheal tube exchanges.

Obese patients may present a challenge during airway management. However, a debate continues to whether morbidly obese patients are more difficult to intubate than the general population. Juvin et al. reported the incidence of difficult intubation to be 15.5% in morbidly obese patients, compared with 2.2% in controls [3]. Gonzalez et al. found the difficult intubation rate of 14.3% in obese patients versus 3% in nonobese patients [4]. In contrast, Ezri et al. and Lundstrøm et al. reported that BMI was weakly associated with difficult intubation in morbidly obese patients, when compared to nonobese patients [5, 6]. Among morbidly obese patients, Brodsky et al. and Neligan et al. demonstrated that increased BMI was not an independent risk factor of difficult intubation [7, 8].

Morbidly obese patients have decreased functional capacity (FRC), increased alveolar-to-arterial (A-a) oxygen gradient [9, 10], and increased oxygen consumption [11]. Therefore, even if airway management—including airway tube exchange—is not difficult, they will desaturate faster than their leaner counterpart after cessation of ventilation. Patients whose airway management is difficult will be even more at risk of desaturation.

Due to concerns of possible difficult airway and/or rapid desaturation after cessation of spontaneous ventilation, some anesthesiologists opt to perform awake fiberoptic intubation (AFI) in morbidly obese patients, especially in those with very large BMIs or with other associated characteristics that predict difficult intubation [12]. When the primary intubation is done with AFI, one misses the opportunity to test the difficulty of mask ventilation, as well as of laryngoscopy and intubation. If one chooses to perform an AFI for primary intubation in the first place because there are concerns that conventional laryngoscopy could be difficult, it will then be illogical to assume that airway exchange with conventional laryngoscopy will be easy.

As for airway management using direct laryngoscopy, it has been demonstrated that, when using direct laryngoscopy for intubation, a “ramped” position or HELP (head-elevated laryngoscopy position: head, shoulders, and upper body elevated so that the suprasternal notch and the external auditory meatus are in the same horizontal imaginary line) provides significantly improved laryngoscopic views in this patient population, when compared to standard sniffing position [13, 14]. Ramping the patient for primary laryngoscopy is generally done by placing blankets, or premanufactured elevation pillow [15], on the surgical table before moving the patient onto it. This ramp will have to be removed for most surgical procedures. When the airway device needs to be exchanged, reinserting the ramp underneath an anesthetized, intubated morbidly obese patient may be very difficult and can lead a loss of airway, as well as injuries to anesthesiologists and OR personnel. When reinsertion of the ramp is not possible, a nondifficult primary intubation in the “ramped” position could turn into a difficult one when the patient is in sniffing position.

From the above examples, when the primary intubation has been successful with other methods other than conventional laryngoscopy in standard sniffing position, one should bear in mind that the tube exchange could be more difficult than the primary intubation.

This paper describes airway tube exchanging techniques, besides conventional direct laryngoscopy, in morbidly obese patients, in order to secure the airway and successfully change between a single-lumen tube (SLT) and a double-lumen tube (DLT) when necessary.

The circumstances in some of the references are either airway exchanging techniques in nonobese patients with other causes of difficult airway or primary intubation techniques in morbidly obese patients. There has not been literature specifically dedicated to airway tube exchanging techniques in morbidly obese patients; therefore some extrapolation is required from the existing evidence.

The examples of situations when airway tubes need to be exchanged include, but are not limited to the following: (1) an LMA needs to be upgraded to an endotracheal tube; (2) an SLT needs to be changed to a DLT for lung isolation (e.g., due to anesthesiologist's preference for lung isolation or failure of bronchial blocker to provide adequate isolation); (3) a DLT needs to be exchanged for a SLT (e.g., postoperative mechanical ventilation is required after intraoperative lung isolation with a DLT).

It should be noted that when lung isolation is required, an SLT does not have to always be exchanged for a DLT. Using a bronchial blocker with the indwelling SLT is another alternative, and the endotracheal tube will not need to be exchanged at all.

## 2. Use of Airway Exchange Catheter

**Figure 1:**
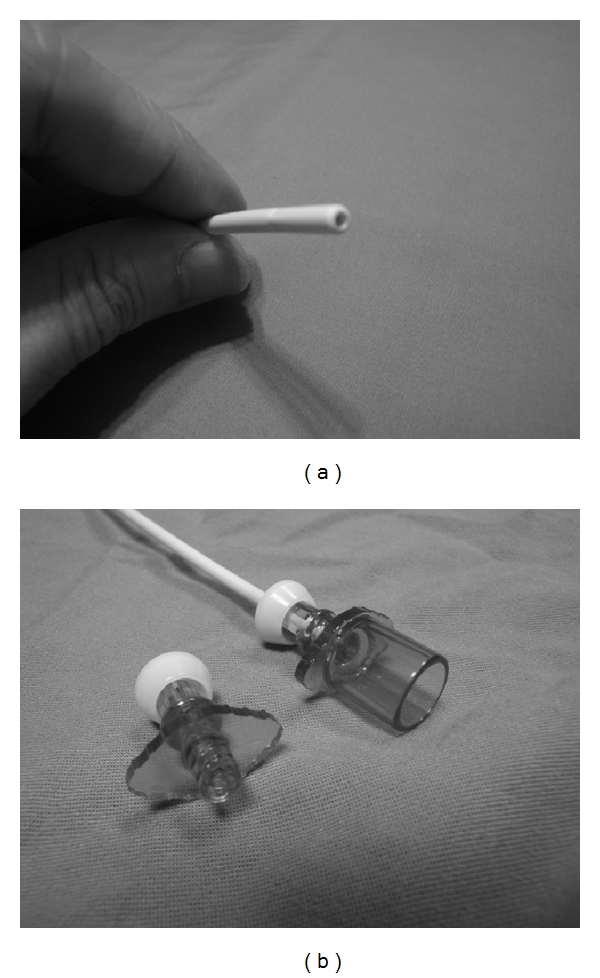
(a) Center hollow channel. (b) Adaptors for oxygen insufflation or jet ventilation.

An airway exchange catheter (AEC) can be used to facilitate exchanging from an SLT to a DLT, or vice versa. This device has a center hollow channel and a universal fit adapter through which oxygen insufflation or jet ventilation can be administered to allow more time for the tube exchanging process (Figure 1). Literature is not available regarding effectiveness of oxygen supplementation via this hollow channel in morbidly obese patients. One could presume that it may not be as effective as in nonobese population as lower lung compliance will reduce oxygen flow via the AEC [16].

 Jet ventilation uses high pressure (10–50 psi), and there have been reports of tension pneumothoraces caused by jet ventilation via the hollow lumen of the AEC in nonmorbidly obese patients [17–19]. Even oxygen insufflations with low driving pressure could potentially cause pneumothorax especially in the presence of upper airway obstruction that impairs the exhalation of the insufflated gas volume [20]. Morbid obesity with obstructive sleep apnea, or simply with redundant tissue in the upper airway, could be one of the examples of such obstruction.

AECs are commercially available in various lengths and sizes, and careful selection to serve the purpose is important. It must be of sufficient length (at least 83 cm long) to ensure tracheal introduction of the DLT [21]. Despite the length required, AECs should not be advanced against resistance or deeper than 24 cm from the lip to avoid airway laceration or perforation [22, 23].

**Figure 2:**
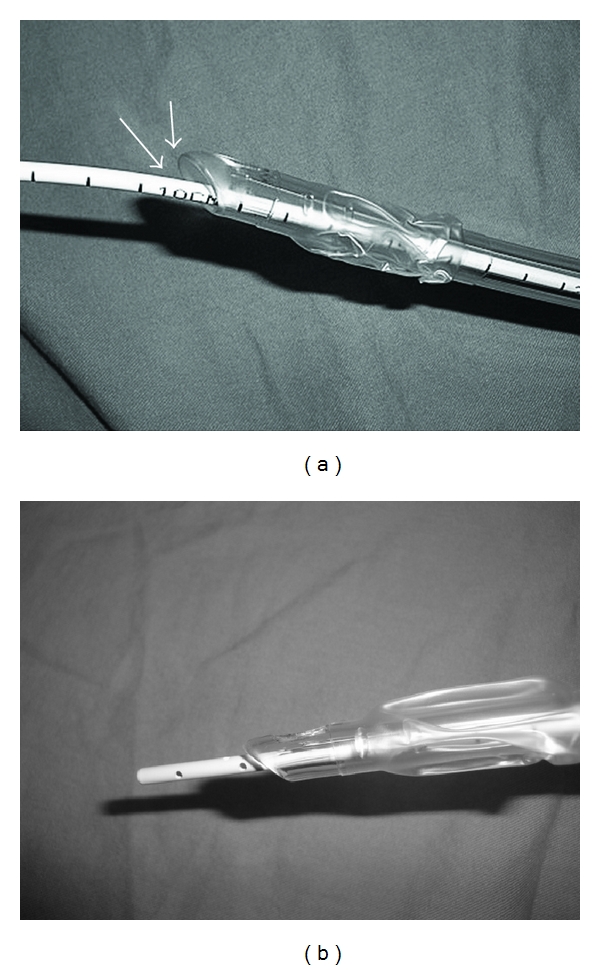
(a) When the OD of the AEC is significantly smaller than the ID of the tube, the tip of the tube protrudes away from the AEC and is susceptible to impingement at the glottis. (b) With the OD of the AEC relatively large compared to the ID of the tube, the tip of the tube does not protrude as much and the possibility of impingement is reduced.

**Figure 3:**
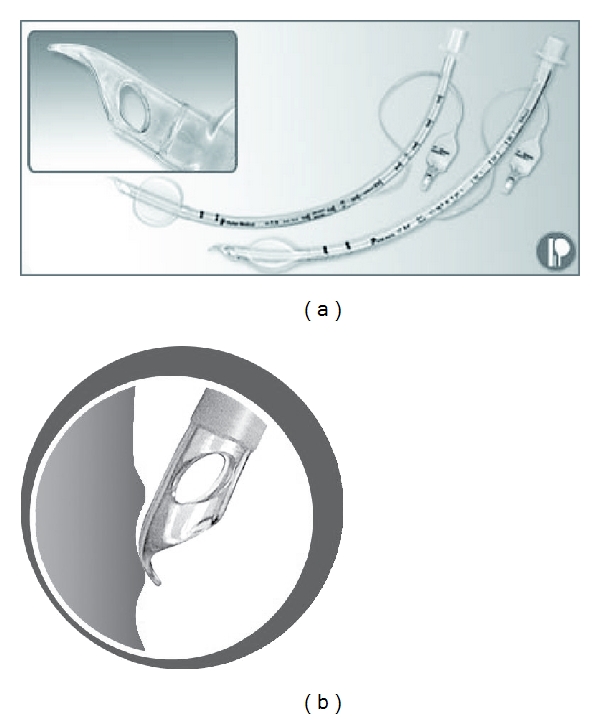
Impingement is less likely with taper-tipped tubes.

**Figure 4:**
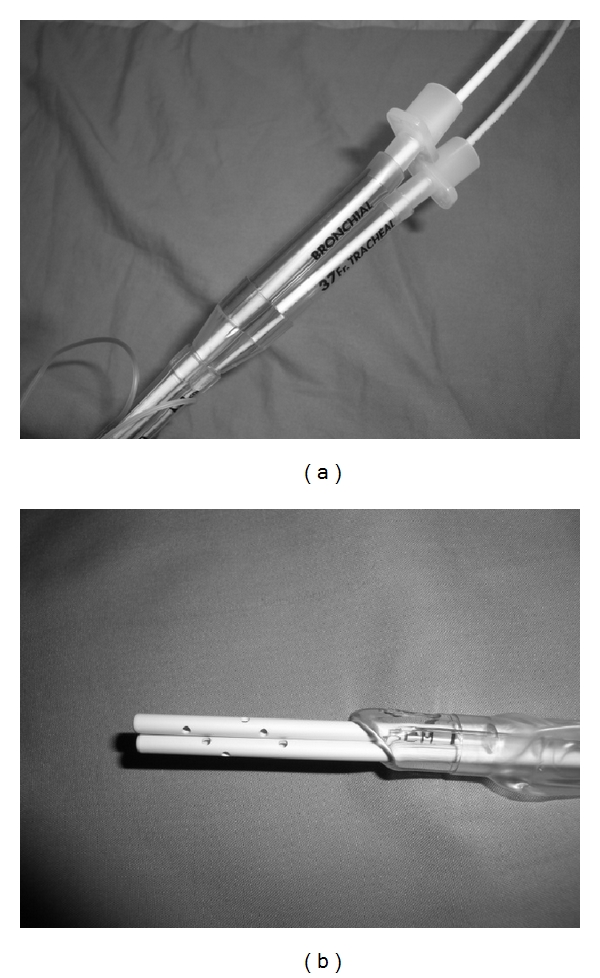
(a) Two AECs used—one inserted into each lumen of the DLT. (b) The combined OD of the 2 AECs is relatively large compared to the ID of the SLT and helps reduce the protrusion of the tip of the tube.

After inserting the AEC into the first (indwelling) tube to the appropriate depth, the tube is then extubated. One should be careful not to pull the AEC out from the trachea along with the first tube. When the second tube (the tube that will be exchanged for) is railroaded over the AEC into the trachea, the tip of the tube may impinge at the glottis and will not advance into the trachea. To reduce the chance of impingement, use an AEC with a relatively large outer diameter (OD) compared to the internal diameter (ID) of the second tube [24, 25] (Figure 2). When changing from a DLT to an SLT, the AEC that fits through one lumen of the DLT (the first tube) will be relatively small compared to the ID of the SLT (the second tube) and will likely result in impingement. A taper-tipped tube (Flex-Tip; Parker Medical, Englewood, CO, USA) (Figure 3) [26] may be useful in this situation [27]. Alternatively, 2 AECs can be used—one through the tracheal lumen and one through the bronchial lumen of the indwelling DLT (Figure 4(a)). The combined OD of the 2 AECs will be relatively large compared to the ID of the SLT that will be railroaded over them and will help reduce the possibility of impingement [28] (Figure 4(b)). The size and fit of the AEC in the DLT and the SLT must be tested in vitro before the exchange.

When impingement does occur, 90° counterclockwise rotation of the tube may help disengage it [29]. Concomitant use of a laryngoscope will help lift the soft tissue—that would otherwise cause the AEC and the second tube to curve—and facilitate passage of the tube over the AEC into the trachea [30, 31].

## 3. Role of Videolaryngoscopes/Optical Laryngoscopes

There has not been literature demonstrating the role of videolaryngoscopes/optical laryngoscopes in facilitating endotracheal tube exchange in morbidly obese patients. However, existing evidence suggests that this type of equipment, by providing visualization of the vocal cords, could be used for that purpose.

There have been case reports of successful endotracheal tube exchange, using such equipment, in nonobese patients with difficult intubation. Smith et al. [32] reported that a WuScope (Achi Corporation, San Jose, CA, USA) helped them to successfully exchange from a DLT to an SLT under vision, without having to use an AEC. Chen et al. [33] used a GlideScope (Saturn Biomedical System Inc, Burnaby, BC, Canada) to exchange from an SLT to a DLT over an AEC. Poon et al. [34] reported successful intubation of a DLT over an AEC using an Airway Scope (Pentax, Tokyo, Japan).

Various types of videolaryngoscopes/optical laryngoscopes have been shown to improve intubation condition in morbidly obese patients [35–39]. This equipment provides visualization of the vocal cords, as well as of the endotracheal tube advancing into the trachea, and could make endotracheal tube exchange with an AEC more secure.

## 4. Summary

Airway management in morbidly obese patients can be challenging. It is important to first secure the airway either with direct laryngoscopy in head-elevated laryngoscopic position, videolaryngoscope, or AFI. When another different type of airway tube is required for one-lung ventilation or for postoperative mechanical ventilation after the conclusion of the surgical procedure, the technique used for the primary intubation may not be feasible in anesthetized morbidly obese patients. Videolaryngoscopes/optical laryngoscopes could be very useful in facilitating airway tube exchange. Nevertheless, use of AECs remains the predominant method of exchange for morbidly obese patients, and it is important for anesthesiologists to be familiar with using these devices.

It should be noted that airway tube exchange with an AEC in morbidly obese patients is not a benign process and should be done only when necessary and with extreme caution. An AEC inserted too deep into the trachea could lacerate the tracheobronchial tree. Due to poor respiratory mechanics and increased oxygen consumption, morbidly obese patients could desaturate quickly during the exchange process. When that happens, mask ventilation with an AEC in place could be difficult. Jet ventilation or oxygen insufflations via the AEC could incur risks of pneumothorax, especially if there is upper airway obstruction that hinders exhalation. The positioning/technique used during the primary intubation may not be feasible during the tube exchange, making the exchange more difficult—a videolaryngoscope/optical laryngoscope could help mitigate this problem. After the first tube is extubated, the second tube may not easily pass over the AEC into the vocal cords, especially if the primary intubation was difficult and traumatic. During the exchange process, one could accidentally pull the AEC from the trachea and end up with an extubated, swollen, difficult airway in a patient who quickly desaturates.
